# Incorporation of CAD (computer-aided detection) with thin-slice lung CT in routine 18F-FDG PET/CT imaging read-out protocol for detection of lung nodules

**DOI:** 10.1186/s41824-023-00177-2

**Published:** 2023-09-18

**Authors:** Ujwal Bhure, Matthäus Cieciera, Dirk Lehnick, Maria del Sol Pérez Lago, Hannes Grünig, Thiago Lima, Justus E. Roos, Klaus Strobel

**Affiliations:** 1 Department of Nuclear Medicine and Radiology, Cantonal Hospital Lucerne, Lucerne, Switzerland; 2 Faculty of Health Sciences and Medicine, University of Lucerne, Frohburgstrasse 3, 6002 Lucerne, Switzerland; 3 Clinical Trial Unit Central Switzerland, University of Lucerne, 6002 Lucerne, Switzerland; 4 Division of Nuclear Medicine, Department of Nuclear Medicine and Radiology, Cantonal Hospital Lucerne, 6000 Lucerne 16, Switzerland

**Keywords:** Lung nodules, CAD, Computer-aided detection, 18F-FDG PET/CT, Thin-slice CT, Thick-slice CT, Lung metastases, Artificial intelligence, Machine learning, Deep learning

## Abstract

**Objective:**

To evaluate the detection rate and performance of 18F-FDG PET alone (PET), the combination of PET and low-dose thick-slice CT (PET/lCT), PET and diagnostic thin-slice CT (PET/dCT), and additional computer-aided detection (PET/dCT/CAD) for lung nodules (LN)/metastases in tumor patients. Along with this, assessment of inter-reader agreement and time requirement for different techniques were evaluated as well.

**Methods:**

In 100 tumor patients (56 male, 44 female; age range: 22–93 years, mean age: 60 years) 18F-FDG PET images, low-dose CT with shallow breathing (5 mm slice thickness), and diagnostic thin-slice CT (1 mm slice thickness) in full inspiration were retrospectively evaluated by three readers with variable experience (junior, mid-level, and senior) for the presence of lung nodules/metastases and additionally analyzed with CAD. Time taken for each analysis and number of the nodules detected were assessed. Sensitivity, specificity, positive and negative predictive value, accuracy, and Receiver operating characteristic (ROC) analysis of each technique was calculated. Histopathology and/or imaging follow-up served as reference standard for the diagnosis of metastases.

**Results:**

Three readers, on an average, detected 40 LN in 17 patients with PET only, 121 LN in 37 patients using ICT, 283 LN in 60 patients with dCT, and 282 LN in 53 patients with CAD. On average, CAD detected 49 extra LN, missed by the three readers without CAD, whereas CAD overall missed 53 LN. There was very good inter-reader agreement regarding the diagnosis of metastases for all four techniques (kappa: 0.84–0.93). The average time required for the evaluation of LN in PET, lCT, dCT, and CAD was 25, 31, 60, and 40 s, respectively; the assistance of CAD lead to average 33% reduction in time requirement for evaluation of lung nodules compared to dCT. The time-saving effect was highest in the less experienced reader. Regarding the diagnosis of metastases, sensitivity and specificity combined of all readers were 47.8%/96.2% for PET, 80.0%/81.9% for PET/lCT, 100%/56.7% for PET/dCT, and 95.6%/64.3% for PET/CAD. No significant difference was observed regarding the ROC AUC (area under the curve) between the imaging methods.

**Conclusion:**

Implementation of CAD for the detection of lung nodules/metastases in routine 18F-FDG PET/CT read-out is feasible. The combination of diagnostic thin-slice CT and CAD significantly increases the detection rate of lung nodules in tumor patients compared to the standard PET/CT read-out. PET combined with low-dose CT showed the best balance between sensitivity and specificity regarding the diagnosis of metastases per patient. CAD reduces the time required for lung nodule/metastasis detection, especially for less experienced readers.

## Background

Lung is a very common site for metastasis from various malignancies. Detection of small, especially subcentimeter sized, lung nodules is an important critical task during routine oncologic whole body 18F-Fluorodeoxyglucose Positron emission tomography/computed tomography (18F-FDG PET/CT) evaluation, especially when these nodules are not FDG avid. Often small lung nodules are early signs for metastasis in cancer patients with important therapeutic impact. FDG uptake is an important criteria for diagnosis of lung metastases in these patients with various malignancies, but not sensitive enough for the detection of small subcentimeter sized and early metastatic lung nodules (Strobel et al. 2007; Volker et al. 2007; Sawicki et al. 2016). Failure to detect small and early cancerous lung lesions on imaging studies might be a reason for malpractice suits (Baker et al. 2013; Whang et al. 2013; Weikert et al. 2019). The reasons of misdiagnosis are multi-layered and include pattern recognition error, incomplete/unsatisfactory search, overload of data, stressed physicians, etc. (Del Ciello et al. 2017). Computer-aided detection (CAD) is commercially available for LN detection since the early 2000s and has been studied a lot in the last decade on dedicated chest CTs with deep inspiration breath-hold technique. Classical machine learning and radiomics have been used for lung nodule detection and segmentation with nodule volumetry and characterization. The more recent rise of deep learning with CNN (convoluted neural network) and availability of large annotated lung nodule datasets have allowed the development of CAD tools with fewer false-positives per scan (Chassagnon et al. 2023).

Technically, by definition, lung nodules are focal opacities, well- or poorly-defined, measuring less than 30 mm in diameter (Hansell et al. 2008). Lung nodule detection is an important task in oncologic PET/CT imaging for metastatic work up, especially in tumors with predilection for lung metastases like melanoma, sarcoma, colorectal, head and neck, and thyroid cancers.

Computed Tomography (CT) represents the current standard for detection of small lung nodules (LN), and dedicated post-processing methods have been established to further increase LN detection (Davis 1991). Besides the detection of LN on PET images due to increased uptake of FDG, the dedicated interpretation of the CT data part—an integral component of any PET/CT examination reading—applying lung window settings, increases the sensitivity for the detection of lung metastases in cancer patients (Strobel et al. 2007; Volker et al. 2007; Sawicki et al. 2016). To precisely detect these lung nodules with PET/CT, low-dose thick-slice CT with shallow breathing, thin-slice full inspiration breath-hold CT, and even respiratory gated PET/CT to reduce respiratory motion related artifacts, have been implemented (Werner et al. 2009; Farid et al. 2015). The effective radiation dose in low-dose chest CT scan is generally about 1.5 mSv (range: 1–5 mSv) while a conventional “normal-dose” diagnostic chest CT scan might result in an effective radiation dose of approximately 8 mSv or more, depending on the specific equipment and protocol used (Coakley et al. 2011). The slice thickness in thick-slice CT usually ranges from 5 to 10 mm and in thin-slice CT it ranges from 1 to 2.5 mm. Additionally, implementation of advanced post-processing methods, such as the use of thin-slice MIP (maximum intensity projection) images and computer-aided detection (CAD) systems, demonstrated a benefit in detection of lung nodules in the chest CT data (Beyer et al. 2007; Peloschek et al. 2007; Kawel et al. 2009; Messay et al. 2010; Roos et al. 2010; Christe et al. 2013). CAD systems were validated in both secondary and primary concurrent reader paradigms. To our knowledge, incorporation of CAD systems for the detection of LN in the routine oncologic whole body 18F-FDG PET/CT imaging protocol have not been validated and implemented. The goal of this study was to compare the performance of 18F-FDG PET, low-dose thick-slice CT, diagnostic thin-slice CT, and CAD as a secondary reader for the detection of lung nodules in tumor patients.

## Methods

The study was approved by the Ethics Committee and the need for written informed consent was waived according to the unique retrospective data analysis design. Consecutive 18F-FDG PET/CT scans of 100 patients (56 male, 44 female; age range: 22–93 years, median age: 63 years) including low-dose CT and diagnostic thin-slice lung CT images were retrospectively selected. Patients had various types of malignancies: melanoma (*n* = 49), head and neck cancer (23), colorectal cancer (8), and the remaining patients (20) with mix of other tumors, such as carcinoma of cervix/uterus, breast, sarcoma, and cholangiocarcinoma. The inclusion criteria were availability of above-mentioned imaging datasets in a 18F-FDG PET/CT examination of these consecutive tumor patients with predilection for lung metastases and follow-up imaging of either 18F-FDG PET/CT or diagnostic chest CT.

### 18F-FDG PET/CT imaging

PET/CT scans were acquired on a Discovery 600 unit (GE Healthcare, USA) from vertex to mid-thigh after intravenous injection of 18F-FDG (18Fluorine-fluorodeoxyglucose) (mean activity 302.5 MBq; range 257–355 MBq). 18F-FDG PET/CT imaging protocol included firstly, a low-dose CT (lCT) with shallow breathing from vertex to mid-thigh with the following parameters: tube voltage 120 kV, tube current: automatic exposure control, pitch 0.88, slice thickness reconstruction in 5 mm; secondly a PET study (2 min acquisition time per bed position); and thirdly, a diagnostic lung CT (dCT) in expiration and breath-hold technique (tube voltage 120 kV, tube current 180 mA, pitch 1.35, slice thickness reconstruction in 1 mm).

### Image interpretation

The read-out was performed by three independent readers with various levels of experience in reading CT and PET/CT images, a senior reader with > 15 years of experience (Reader 1), a mid-level reader with 10 years of experience (Reader 2), and a junior reader with 1 year of experience (Reader 3). Each study was retrieved from picture archiving and communication system—PACS (Merlin PACS, Phönix-PACS, Freiburg, Germany) and loaded onto GE ADW workstation (GE Healthcare, USA), wherein analysis of the scans was done. PET images were evaluated for the presence of LN. Lung nodules were defined as focal visible uptake of FDG in the lungs. In CT, lung nodules were defined in visual assessment as round opacities, well- or poorly-defined, measuring less than 3 cm in diameter. Triangular and calcified nodules were excluded from the analysis, as these often represent benign findings, such as intrapulmonary lymph nodes (Hansell et al. 2008). Thin-slice lung CT images were evaluated for the presence of LN by scrolling through maximum intensity projection (MIP) images. Thin-slice lung CT images in full inspiration were loaded into the CAD software (Lung VCAR, GE Healthcare, Chicago, IL, USA) for computer-aided detection of lung nodules (Chen et al. 2012). Lung VCAR software uses innovative Digital Contrast Agent (DCA) feature (a 3D filter), which automatically highlights spherical shapes to enhance visualization of suspicious lung nodules. A threshold of 2 mm was used for the software evaluation. Default number of suspicious nodules highlighted by the CAD software were noted (CAD primary reading—CADp). CADp detected lesions were checked and filtered by the physician and obvious false positive markings due to vessel crossings, artefacts, or benign nodules with calcifications were not considered as positive findings (CAD secondary reading—CADs). The order of the reading of the 4 different image datasets was random to reduce any recall bias. The time taken by each reader for evaluation of lung nodules for each modality was noted along with the number of nodules identified by the reader. Follow-up scans with either 18F-FDG PET/CT or CT chest were available in all the cases. Based on the nodule morphology, FDG uptake (wherever applicable), follow-up imaging, and clinical information, consensus opinion among the three readers was built up on the probable benign or metastatic nature of the nodules and served as ‘reference standard’.

### Statistics

The statistical analyses were performed using Stata (version 17.0, StataCorp, College Station, Texas, USA). Categorical variables were summarized by absolute and relative frequencies. Quantitative variables were analyzed using descriptive statistics. In order to assess the inter-reader agreement for the different techniques with regard to the judgment on whether a patient was considered to have metastases (yes/no), Cohen/Conger’s kappa coefficients and corresponding 95% confidence intervals were calculated. To compare the different techniques with regard to their potential to discriminate between patients with and without metastases, diagnostic metrics such as sensitivity, specificity, negative predictive value (NPV), positive predictive value (PPV), and accuracy were determined as well as ROC AUCs (area under the receiver operating characteristic curve) together with their 95% confidence intervals, overall and by each reader.

## Results

### Nodule detection

**Fig. 1:**
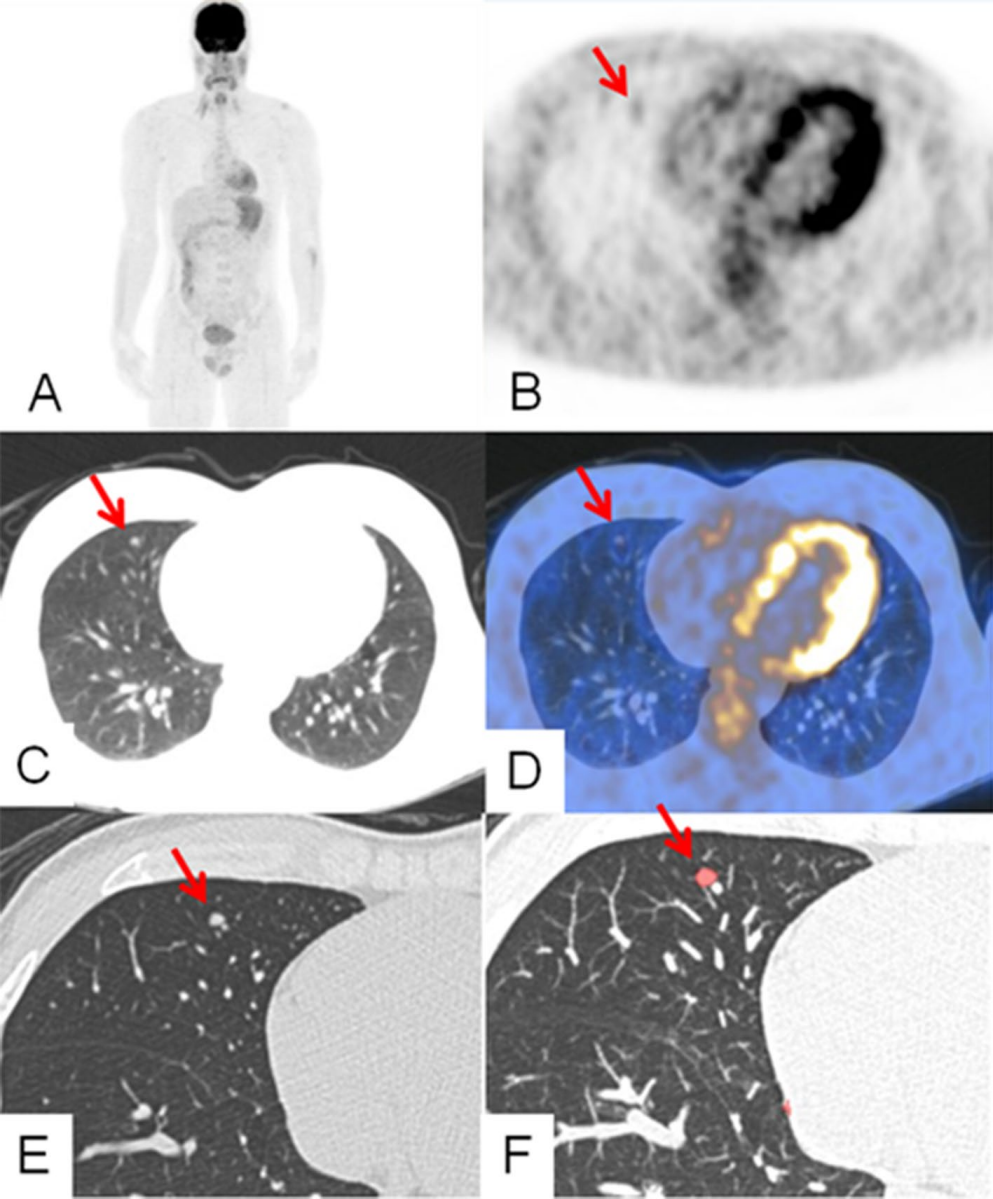
18F-FDG PET/CT images for staging of a 30-year-old male patient after resection of melanoma (Breslow 3.7 mm) around the ear. On MIP (maximum intensity projection) (**A**), axial PET (**B**), and axial fused PET/CT (**D**) images FDG uptake (arrow) is visible in a solitary small nodule in the middle lobe. The nodule (arrow) was detected with low-dose CT (**C**), thin-slice diagnostic CT (**E**), and CAD (**F**). The nodule was resected and was metastatic on histopathology

**Fig. 2:**
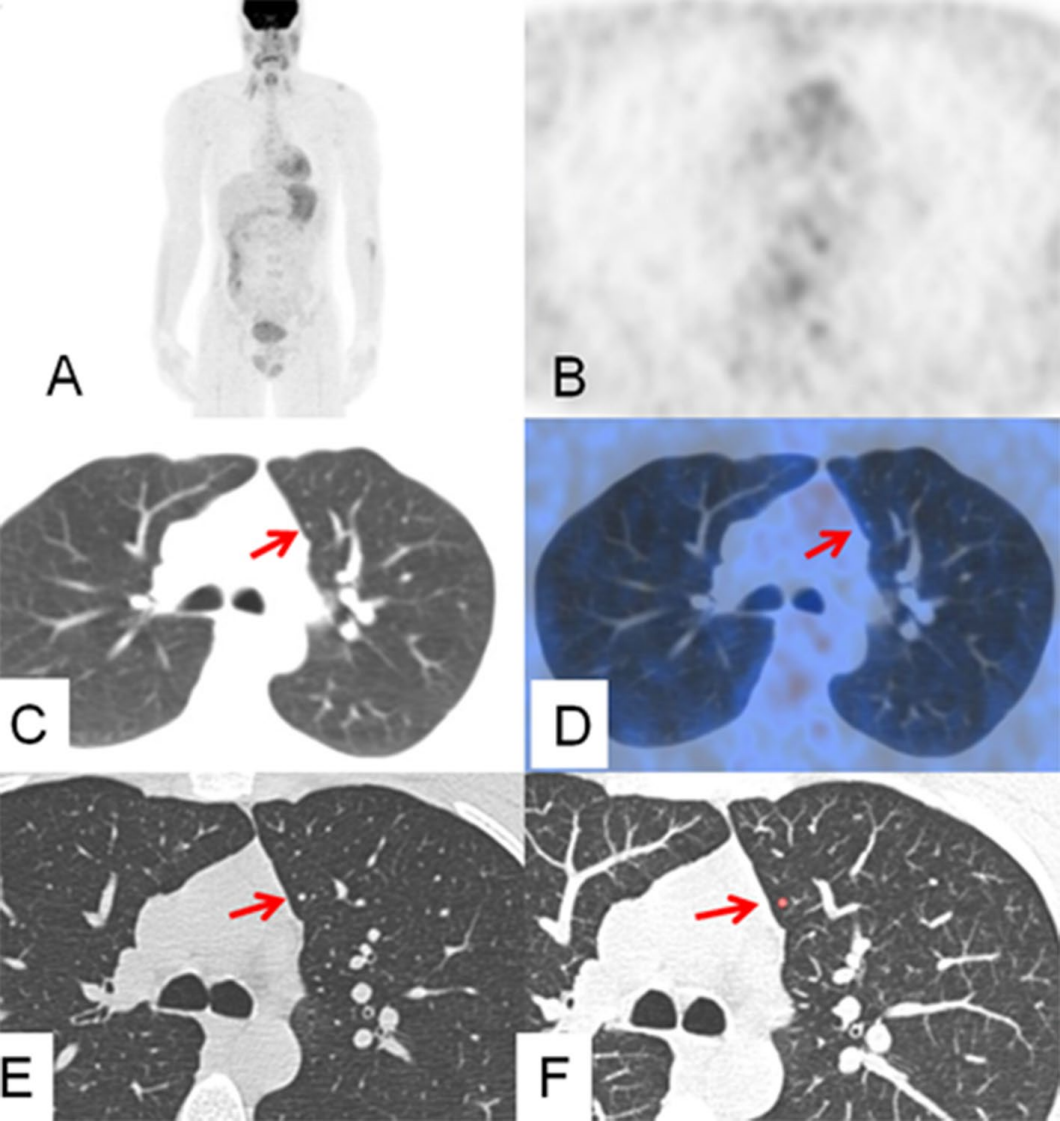
Images of the same patient showing a small nodule in low-dose CT (**C**) and thin-slice diagnostic CT (**E**) (arrow) in the upper left lobe without visible uptake in MIP (**A**), PET (**B**) and PET/CT (**D**) images. The nodule was missed by the readers in all CT images and only detected with CAD (**F**, arrow)

The number of nodules detected by the three readers with four different techniques in the 100 patients, and the time taken for each LN reading are shown in Table 1. On an average, 40 LN were detected in 17 patients using the 18F-FDG PET images only, 121 LN in 37 patients using ICT (Fig. 1), 283 LN in 60 patients with dCT, and 282 LN in 53 patients with dCT using the help of CADs (Fig. 2) (Table 1).

**Table 1:** Performance of three readers with regards to the reading time required and number of nodules detected using different imaging techniques

	Reader 1	Reader 2	Reader 3	Average
Experience	> 15 years	10 years	1 year	
Mean reading time in seconds				
FDG PET	16.42	17.53	40.94	24.96
lCT	23.14	23.85	45.62	30.87
dCT	39.61	43.6	96.58	59.93
CADs	33.61	27.78	58.81	40.07
Number of nodules detected				
FDG PET	39	42	41	40.67
lCT	112	130	121	121
dCT	275	300	276	283.67
CADs	292	306	249	282.33
Extra nodules by CAD	62	54	32	49.33
Nodules missed by CAD	45	52	62	53
Time for CADp	8.3	8.3	8.3	8.3
CADp nodules	1967	1967	1967	1967
Accepted nodules	292	306	249	282.33
Rejected nodules (Fps)	1678	1661	1718	1685.67
FP rate per scan	16.8	16.6	17.2	16.8

*FDG PET* 18F-fluorodeoxyglucose positron emission tomography, *lCT* Low-dose computed tomography, *dCT* diagnostic lung CT, *CADp* Computer-aided detection (primary reading), *CADs* Computer-aided detection (secondary reading), *FP* False positive

On an average, CAD detected 49 extra LN, missed by the three readers, in 100 PET/CT examinations, whereas CAD overall missed nearly 53 LN, which were detected with dCT. The common reasons for CAD missing LN were proximity to vessel, subpleural location or attachment to pleura, or too small a size (2 mm).

The mean number of false-positive LN for CADp were 16.8 per scan. In the given 100 patients, CADp showed total of 1967 nodules, of which on average 282 were accepted by the three readers, and remaining average 1685 were rejected as false markings.

The sizes of the nodules ranged from 2 to 26 mm with a mean size of 4.1 mm (no nodule larger than 30 mm). The nodules were randomly spread across various lobes of the lungs, with more nodules being in peripheral location (78% in peripheral location and 22% in central location) and in lower zones (67% in the lower zones and 33% in the upper zones) (Table 2).

**Table 2:** General distribution of 300 detected lung nodules

Localization of lung nodules	Number (percentage)
Peripheral	233 (78%)
Central	67 (22%)
Right lung	153 (51%)
Left lung	147 (49%)
RUL (right upper lobe)	57 (19%)
ML (middle lobe)	40 (13%)
RLL (right lower lobe)	56 (19%)
LUL (left upper lobe)	41 (14%)
Lingula	21 (7%)
LLL (left lower lobe)	85 (28%)
Upper zones (RUL + LUL)	98 (33%)
Lower zones (RML + RLL + Lingula + LLL)	202 (67%)

### Time requirement for image analysis

The average time for all three readers required for the evaluation of PET, lCT, dCT, and CADs was 25, 31, 60, and 40 s, respectively. Thus, nearly 33% reduction in time requirement for evaluation of lung nodules was achieved with the help of CAD compared to dCT. The maximum benefit was seen for the junior-most reader with approximately 39% of time reduction (details as given in Table 1).

### Inter-reader agreement

There was very good inter-reader agreement (inter-rater reliability) with kappa ranging between 0.84 and 0.93 for four different techniques among the three readers (Table 3).

**Table 3:** Inter-reader agreement for different imaging techniques

	Kappa	95% confidence interval
FDG PET	0.93	(0.85, 1.00)
lCT	0.84	(0.75, 0.93)
dCT	0.85	(0.76, 0.93)
CADs	0.91	(0.84, 0.97)

*FDG PET* 18F-fluorodeoxyglucose positron emission tomography

*lCT* low-dose computed tomography, *dCT* diagnostic lung CT, *CADp* Computer-aided detection

### Performance for diagnosis of lung metastases

Follow-up scans (either 18F-FDG PET/CT or chest CT) were available in all patients. Average follow-up duration was 25.53 months (range: 1–72 months). Interobserver consensus was built up on the true or false nodules. Reference standard for the diagnosis of metastasis was based on the histopathology (*n* = 5) and/or the follow-up imaging (*n* = 100; 18F-FDG PET/CT in 93 and chest CT in 7) and clinical information (all). For example, in cases of increasing sizes of lung nodules with typical morphology in follow-up imaging, were rated as positive for metastasis.

**Fig. 3:**
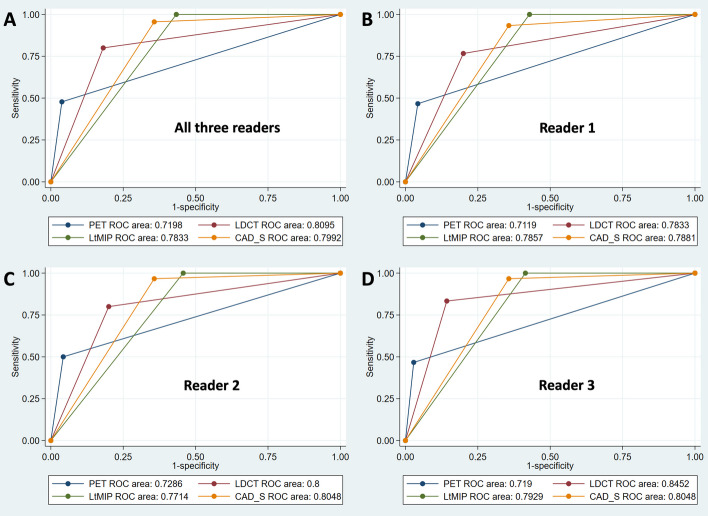
ROC AUC (area under the receiver operating characteristic curve) analysis for all readers regarding the diagnosis of lung metastases on a per patient basis [**A**: for all three readers combined, **B**: for reader 1 (R1), **C**: for reader 2 (R2), **D**: for reader 3 (R3)]

AUC in ROC analysis, sensitivity, specificity, negative predictive value (NPV), positive predictive value (PPV) and accuracy of different readers and different techniques are given in Tables 4 and 5, and Fig. 3.

**Table 4:** Sensitivity, specificity, NPV, PPV, and Accuracy for the diagnosis of lung metastases per patient with different techniques

	R1	R2	R3
**FDG PET** (all values in percentages) (95% confidence interval in brackets)			
Sensitivity	47 (28, 66)	50 (31, 69)	47 (28, 66)
Specificity	96 (88, 99)	96 (88, 99)	97 (90, 100)
NPV*	81 (71, 89)	82 (72, 89)	81 (71, 89)
PPV*	82 (57, 96)	83 (59, 96)	88 (62, 98)
Accuracy*	81 (72, 88)	82 (73, 89)	82 (73, 89)
**FDG PET/lCT** (all values in percentages) (95% confidence interval in brackets)			
Sensitivity	77 (58, 90)	80 (61, 92)	83 (65, 94)
Specificity	80 (69, 89)	80 (69, 89)	86 (75, 93)
NPV*	89 (78, 95)	90 (80, 96)	92 (83, 97)
PPV*	62 (45, 78)	63 (46, 78)	71 (54, 85)
Accuracy*	79 (70, 87)	80 (71, 87)	85 (76, 91)
**FDG PET/dCT** (all values in percentages) (95% confidence interval in brackets)			
Sensitivity	100 (88, 100)	100 (88, 100)	100 (88, 100)
Specificity	57 (45, 69)	54 (42, 66)	59 (46, 70)
NPV*	100 (91, 100)	100 (91, 100)	100 (91, 100)
PPV*	50 (37, 63)	48 (35, 61)	51 (37, 64)
Accuracy*	70 (60, 79)	68 (58, 77)	71 (61, 80)
**FDG PET/CAD** (all values in percentages) (95% confidence interval in brackets)			
Sensitivity	93 (78, 99)	97 (83, 100)	97 (83, 100)
Specificity	64 (52, 75)	64 (52, 75)	64 (52, 75)
NPV*	96 (85, 99)	98 (88, 100)	98 (88, 100)
PPV*	53 (39, 67)	54 (40, 67)	54 (40, 67)
Accuracy*	73 (63, 81)	74 (64, 82)	74 (64, 82)

	FDG PET	PET/lCT	PET/dCT	PET/CAD
**All three readers combined** (all values in percentages) (95% confidence interval in brackets)				
Sensitivity	48 (37, 59)	80 (70, 88)	100 (96, 100)	96 (89, 99)
Specificity	96 (93, 98)	82 (76, 87)	57 (50, 63)	64 (57, 71)
NPV*	81 (76, 86)	91 (85, 94)	100 (97, 100)	97 (93, 99)
PPV*	84 (71, 93)	65 (56, 74)	50 (42, 57)	53 (45, 61)
Accuracy*	82 (77, 86)	81 (76, 86)	70 (64, 75)	74 (68, 79)

^*^based on the 30% prevalence observed in this sample

R1: Reader 1, R2: Reader 2, R3: Reader 3

*FDG PET* 18F-fluorodeoxyglucose positron emission tomography

*lCT *low-dose computed tomography, *dCT* diagnostic lung CT, *CAD* Computer-aided detection

**Table 5:** ROC AUC Analysis for four different imaging techniques for diagnosis of lung metastases

	AUC	95% confidence interval
**Reader 1**		
FDG PET	0.712	(0.618, 0.806)
lCT	0.783	(0.693, 0.874)
dCT	0.786	(0.727, 0.844)
CADs	0.788	(0.716, 0.861)
**Reader 2**		
FDG PET	0.729	(0.634, 0.823)
lCT	0.800	(0.713, 0.887)
dCT	0.771	(0.713, 0.830)
CADs	0.805	(0.739, 0.870)
**Reader 3**		
FDG PET	0.719	(0.626, 0.812)
lCT	0.845	(0.766, 0.925)
dCT	0.793	(0.735, 0.851)
CADs	0.805	(0.739, 0.870)
**Overall, with all three readers combined:**		
FDG PET	0.720	(0.666, 0.773)
lCT	0.810	(0.760, 0.859)
dCT	0.783	(0.750, 0.817)
CADs	0.799	(0.760, 0.838)

ROC AUC Analysis (area under the receiver operating characteristic curve) for four different imaging techniques for diagnosis of lung metastases

*PET* 18F-FDG positron emission tomography, *lCT* low-dose CT, *dCT* diagnostic lung CT, *CADs* Computer-aided detection (secondary reading)

In summary, regarding the diagnosis of metastasis on a patient basis, PET AUC (0.72) was inferior to lCT, dCT, and CAD because of low sensitivity (48%) in all three readers, but lCT, dCT, and CAD showed comparable good results in all the readers (AUC between 0.78 and 0.81). There was no significant difference in the performance of the three readers with regards to the detection of number of LN; however the least experienced reader (Reader 3) required 133% extra time compared to Reader 1 or 2 in evaluation of 18F-FDG PET images, 91% extra time for lCT, 121% extra time for dCT, and 74% extra time for CADs. There was no superiority of the advanced dCT or CAD techniques regarding the diagnosis of metastasis.

## Discussion

The present study demonstrates the feasibility of implementation of CAD software in routine clinical workflow for the detection of lung nodules and metastases in the CT part of 18F-FDG PET/CT studies of tumor patients. To our knowledge, no study in the past has evaluated CAD for this specific purpose in routine 18F-FDG PET/CT read-out protocol. A few studies evaluated successfully CAD like software applications in detection of pulmonary lesions in the PET part of 18F-FDG PET/CT images (Ballangan et al. 2011, 2013; Yang et al. 2014; Cui et al. 2015). In these studies, lung lesions were detected from PET images alone since the image quality of the CT part used for attenuation correction was insufficient for diagnostic evaluation. There is a large variety of PET/CT protocols used nowadays in tumor patients worldwide ranging from ungated PET and low-dose CT as the most simple, to gated PET/CT and diagnostic CT as the most advanced protocols (Werner et al. 2009; Farid et al. 2015). It has been shown that implementation of thin-slice CT can improve the detection of lung nodules and metastases in tumor patients (Strobel et al. 2007). The interpretation of additionally acquired dCT (diagnostic 1 mm thin-slice CT) images with breath-hold in full inspiration increased the detection rate of LN in our study by 20% compared to ICT (low-dose 5 mm thick-slice slice CT) with shallow breathing. PET with CADs performed equally to dCT. Several studies have shown the potential role of CAD software in lung nodule detection in CT alone without PET (Armato et al. 2002; Awai et al. 2004; Peldschus et al. 2005; Roos et al. 2010; Christe et al. 2013). Christe et al. found that the combination of a human observer with a CAD system provides optimal sensitivity for lung nodule detection (Christe et al. 2013). A study by Peldschus et al. has shown that radiologists missed clinically significant lung nodules in 33% of the patients during routine interpretation of the chest scans, emphasizing the use of CAD (Peldschus et al. 2005). Various reconstruction parameters like slice thickness and slice increment can influence the performance of the CAD software. CAD software performs significantly better with thinner slices (Kim et al. 2005; Marten et al. 2005; Gurung et al. 2006). Hence, for the successful implementation of CAD in 18F-FDG PET/CT reading, thin-slice (1 mm) breath-hold CT should be obtained with the PET/CT acquisition protocol. It has been shown that the sensitivity of a single reader plus CAD is higher than the combined reading of two radiologists (Rubin et al. 2005; White et al. 2008). CAD software performance is not influenced by caseload, fatigue, or other factors. In whole body PET/CT interpretation, there is a high chance of missing small lung nodules due to exhaustion and overload of data. Incorporation of CAD into the PET/CT read-out protocol facilitates the detection of lung nodules as the software very clearly highlights the nodules and missing rate is negligible.

One limitation of the available CAD software algorithms is that they are still generating many false-positive (FP) detections, which fall into two categories: a) true nodules with a low probability of malignancy (pleural thickening, partially calcified granulomas, apical scars, thickened walls of emphysema bullae) and b) false nodules (intersection of bronchial or vascular structures and peribronchial thickening). In order to maintain a diagnostically justifiable specificity, the number of FP results has to be reduced by human cross-checking and rejection, respectively. In the present study, CADp produced a mean number of 16.8 false positive nodules per scan.

Teramoto et al. proposed an improved ensemble method for reduction of false-positives using convolutional neural networks, a type of deep learning architecture, using both the CT and PET components (shape and metabolic feature analysis), dramatically helping to improve the results with elimination of false-positives while maintaining the value of true-positives (LeCun et al. 2015; Teramoto et al. 2016). The initial sensitivity in nodule detection was 97.2% with 72.8 false-positives (FP) per case. After incorporating the proposed new FP-reduction method, the false-positives dropped to 4.9 FPs/case, maintaining the sensitivity of detection at 90.1%. Inclusion of the information obtained from the PET component is equally important and the future studies with CAD and artificial intelligence (AI) should include CT as well as PET features for maximization of the output and benefits with acceptable implementation and utilization in the clinical practice.

Interestingly, Liang et al. found a higher probability (though not statistically significant) of detection of nodules in lower lobes, whereas Weikert et al. did not find any such dependency of lesion detection on the location within the lung (Liang et al. 2016; Weikert et al. 2019). In our study, nearly two thirds (67%) of the nodules were in the lower zones (bilateral lower lobes + right middle lobe + lingula) compared to the upper zones (bilateral upper lobes), which showed remaining 33% of the nodules.

Vassallo et al. (2019) compared unassisted and CAD-assisted detection and time efficiency of radiologists in reporting lung nodules on CT scans of patients with extra-thoracic malignancies and found that CAD-assisted reading improved the detection of lung nodules, slightly increasing the reading time. They observed that the total scan reading time increased by 11% using CAD (296 s vs. 329 s). In our study, the average time required for the evaluation of lung nodules in 18F-FDG PET, lCT, dCT, and CADs was 25, 31, 60, and 40 s, respectively, and we could observe a nearly 33% reduction in time requirement for evaluation of lung nodules with the help of CAD compared to dCT. The maximum benefit was demonstrated for the most unexperienced reader with approximately 39% reduction in time requirement for assessment of LN.

Marco Das et al. observed that CAD was especially helpful for detecting small lung nodules and improved the performance of the radiologists, and there was increased agreement among radiologists with the use of the CAD systems (Das et al. 2006). In our study, there was very good interobserver agreement (inter-rater reliability) with kappa ranging between 0.842 and 0.929 for four different techniques among the three readers (*p* < 0.001).

We found an improved detection rate with 1 mm thin-slice lung CT. The lCT (low-dose 5 mm lung CT) could detect 121 LN in 37 patients, whereas dCT (diagnostic 1 mm thin-slice lung CT) could detect 283 LN in 60 patients, nearly 58% more nodules being detected with dCT. Detection of additional nodules without visible FDG uptake, even if related to small size of the lung nodule, might result in recommendation of a short time follow-up scan to exclude or confirm metastatic disease. In our follow-up, we observed that the number and sizes of the nodules were essentially stable in 70 patients and progressed (metastatic nature) in 30 patients. Regarding the diagnosis of lung metastases on a per patient basis, there was no significant difference in the performance of PET/lCT, PET/dCT, and PET/CAD despite of variable reader experience. Though, there was no significant difference in the performance of the three readers with regards to the detection of number of LN, the least experienced reader (Reader 3) required 133% extra time compared to Reader 1 or 2 in evaluation of 18F-FDG PET images, 91% extra time for lCT, 121% extra time for dCT, and 74% extra time for CADs. Means, least experienced reader (Reader 3) took significantly more time for detection of same number of nodules. Reader 3 would have missed more nodules had there been time limit.

PET combined with low-dose CT (PET/lCT) showed the best balance between sensitivity and specificity regarding the diagnosis of metastases per patient. Detection of additional small nodules without visible FDG uptake might prompt the recommendation of a short time follow-up scan to exclude or confirm metastatic disease. Probably, the tiny nodules (less than 5 mm) detected with dCT and CAD may not always be of metastatic nature. The PET and lCT may detect less nodules compared to dCT and CAD, but the nodules detected by them are more likely to be of metastatic nature than those detected by dCT and CAD. The clinical relevance of detecting smaller subcentimeter sized FDG non-avid LN and its impact on outcome has to be shown in further studies.

We believe that the implementation of CAD, AI, and deep learning in the detection of LN by integrating PET, diagnostic CT data, and clinical information has an interesting potential especially in patients with high risk for pulmonary metastases like melanoma, sarcoma, head and neck cancer, and rectal cancer, among others.

## Conclusion

Implementation of CAD for the detection of lung nodules/metastases in routine 18F-FDG PET/CT read-out is feasible. The combination of diagnostic thin-slice CT and CAD significantly increases the detection rate of lung nodules in tumor patients compared to the standard 18F-FDG PET/CT read-out. PET combined with low-dose CT showed the best balance between sensitivity and specificity regarding the diagnosis of metastases per patient. CAD reduces the time required for lung nodule/metastasis detection, especially for less experienced readers.

## Data Availability

The datasets can be made available from the corresponding author on reasonable request.

## Abbreviations

PET: Positron emission tomography
FDG: Fluorodeoxyglucose
CT: Computed tomography
lCT: Low-dose (thick-slice) CT
dCT: Diagnostic (thin-slice) CT
CAD: Computer-aided detection
CADp: CAD primary reading
CADs: CAD secondary reading
LN: Lung nodules
MIP: Maximum intensity projection
ROC: Receiver operating characteristic
AUC: Area under the curve
ROC AUC: Area under the receiver operating characteristic curve
PPV: Positive predictive value
NPV: Negative predictive value
FP: False positive
AI: Artificial intelligence

## References

[CR1] Armato SG, Li F, Giger ML, Macmahon H, Sone S, Doi K (2002). Lung cancer: performance of automated lung nodule detection applied to cancers missed in a CT screening program. Radiology.

[CR2] Awai K, Murao K, Ozawa A, Komi M, Hayakawa H, Hori S, Nishimura Y (2004). Pulmonary nodules at chest CT: effect of computer-aided diagnosis on radiologists' detection performance. Radiology.

[CR3] Baker SR, Patel RH, Yang L, Lelkes VM, Castro A (2013). Malpractice suits in chest radiology: an evaluation of the histories of 8265 radiologists. J Thorac Imaging.

[CR4] Ballangan C, Wang X, Fulham M, Eberl S, Yin Y, Feng D (2011). Automated delineation of lung tumors in PET images based on monotonicity and a tumor-customized criterion. IEEE Trans Inf Technol Biomed.

[CR5] Ballangan C, Wang X, Fulham M, Eberl S, Feng DD (2013). Lung tumor segmentation in PET images using graph cuts. Comput Methods Progr Biomed.

[CR6] Beyer F, Zierott L, Fallenberg EM, Juergens KU, Stoeckel J, Heindel W, Wormanns D (2007). Comparison of sensitivity and reading time for the use of computer-aided detection (CAD) of pulmonary nodules at MDCT as concurrent or second reader. Eur Radiol.

[CR7] Chassagnon G, De Margerie-Mellon C, Vakalopoulou M, Marini R, Hoang-Thi TN, Revel MP, Soyer P (2023). Artificial intelligence in lung cancer: current applications and perspectives. Jpn J Radiol.

[CR8] Chen B, Barnhart H, Richard S, Colsher J, Amurao M, Samei E (2012). Quantitative CT: technique dependence of volume estimation on pulmonary nodules. Phys Med Biol.

[CR9] Christe A, Leidolt L, Huber A, Steiger P, Szucs-Farkas Z, Roos JE, Heverhagen JT, Ebner L (2013). Lung cancer screening with CT: evaluation of radiologists and different computer assisted detection software (CAD) as first and second readers for lung nodule detection at different dose levels. Eur J Radiol.

[CR10] Coakley FV, Gould R, Yeh BM, Arenson RL (2011). CT radiation dose: what can you do right now in your practice? AJR. Am J Roentgenol.

[CR11] Cui H, Wang X, Zhou J, Eberl S, Yin Y, Feng D, Fulham M (2015). Topology polymorphism graph for lung tumor segmentation in PET-CT images. Phys Med Biol.

[CR12] Das M, Muhlenbruch G, Mahnken AH, Flohr TG, Gundel L, Stanzel S, Kraus T, Gunther RW, Wildberger JE (2006). Small pulmonary nodules: effect of two computer-aided detection systems on radiologist performance. Radiology.

[CR13] Davis SD (1991). CT evaluation for pulmonary metastases in patients with extrathoracic malignancy. Radiology.

[CR14] Del Ciello A, Franchi P, Contegiacomo A, Cicchetti G, Bonomo L, Larici AR (2017). Missed lung cancer: when, where, and why?. Diagn Interv Radiol.

[CR15] Farid K, Poullias X, Alifano M, Regnard JF, Servois V, Caillat-Vigneron N, Petras S (2015). Respiratory-gated imaging in metabolic evaluation of small solitary pulmonary nodules: 18F-FDG PET/CT and correlation with histology. Nucl Med Commun.

[CR16] Gurung J, Maataoui A, Khan M, Wetter A, Harth M, Jacobi V, Vogl TJ (2006). Automated detection of lung nodules in multidetector CT: influence of different reconstruction protocols on performance of a software prototype. RoFo Fortschritte Auf Dem Gebiete Der Rontgenstrahlen Und Der Nuklearmedizin.

[CR17] Hansell DM, Bankier AA, Macmahon H, Mcloud TC, Muller NL, Remy J (2008). Fleischner Society: glossary of terms for thoracic imaging. Radiology.

[CR18] Kawel N, Seifert B, Luetolf M, Boehm T (2009). Effect of slab thickness on the CT detection of pulmonary nodules: use of sliding thin-slab maximum intensity projection and volume rendering. AJR Am J Roentgenol.

[CR19] Kim JS, Kim JH, Cho G, Bae KT (2005). Automated detection of pulmonary nodules on CT images: effect of section thickness and reconstruction interval–initial results. Radiology.

[CR20] Lecun Y, Bengio Y, Hinton G (2015). Deep learning. Nature.

[CR21] Liang M, Tang W, Xu DM, Jirapatnakul AC, Reeves AP, Henschke CI, Yankelevitz D (2016). Low-dose CT screening for lung cancer: computer-aided detection of missed lung cancers. Radiology.

[CR22] Marten K, Grillhosl A, Seyfarth T, Obenauer S, Rummeny EJ, Engelke C (2005). Computer-assisted detection of pulmonary nodules: evaluation of diagnostic performance using an expert knowledge-based detection system with variable reconstruction slice thickness settings. Eur Radiol.

[CR23] Messay T, Hardie RC, Rogers SK (2010). A new computationally efficient CAD system for pulmonary nodule detection in CT imagery. Med Image Anal.

[CR24] Peldschus K, Herzog P, Wood SA, Cheema JI, Costello P, Schoepf UJ (2005). Computer-aided diagnosis as a second reader: spectrum of findings in CT studies of the chest interpreted as normal. Chest.

[CR25] Peloschek P, Sailer J, Weber M, Herold CJ, Prokop M, Schaefer-Prokop C (2007). Pulmonary nodules: sensitivity of maximum intensity projection versus that of volume rendering of 3D multidetector CT data. Radiology.

[CR26] Roos JE, Paik D, Olsen D, Liu EG, Chow LC, Leung AN, Mindelzun R, Choudhury KR, Naidich DP, Napel S, Rubin GD (2010). Computer-aided detection (CAD) of lung nodules in CT scans: radiologist performance and reading time with incremental CAD assistance. Eur Radiol.

[CR27] Rubin GD, Lyo JK, Paik DS, Sherbondy AJ, Chow LC, Leung AN, Mindelzun R, Schraedley-Desmond PK, Zinck SE, Naidich DP, Napel S (2005). Pulmonary nodules on multi-detector row CT scans: performance comparison of radiologists and computer-aided detection. Radiology.

[CR28] Sawicki LM, Grueneisen J, Buchbender C, Schaarschmidt BM, Gomez B, Ruhlmann V, Umutlu L, Antoch G, Heusch P (2016). Evaluation of the outcome of lung nodules missed on 18F-FDG PET/MRI compared with 18F-FDG PET/CT in patients with known malignancies. J Nucl Med Off Publ Soc Nucl Med.

[CR29] Strobel K, Dummer R, Husarik DB, Perez Lago M, Hany TF, Steinert HC (2007). High-risk melanoma: accuracy of FDG PET/CT with added CT morphologic information for detection of metastases. Radiology.

[CR30] Teramoto A, Fujita H, Yamamuro O, Tamaki T (2016). Automated detection of pulmonary nodules in PET/CT images: ensemble false-positive reduction using a convolutional neural network technique. Med Phys.

[CR31] Vassallo L, Traverso A, Agnello M, Bracco C, Campanella D, Chiara G, Fantacci ME, Lopez Torres E, Manca A, Saletta M, Giannini V, Mazzetti S, Stasi M, Cerello P, Regge D (2019). A cloud-based computer-aided detection system improves identification of lung nodules on computed tomography scans of patients with extra-thoracic malignancies. Eur Radiol.

[CR32] Volker T, Denecke T, Steffen I, Misch D, Schonberger S, Plotkin M, Ruf J, Furth C, Stover B, Hautzel H, Henze G, Amthauer H (2007). Positron emission tomography for staging of pediatric sarcoma patients: results of a prospective multicenter trial. J Clin Oncol Off J Am Soc Clin Oncol.

[CR33] Weikert T, Akinci D'antonoli T, Bremerich J, Stieltjes B, Sommer G, Sauter AW (2019). Evaluation of an AI-powered lung nodule algorithm for detection and 3D segmentation of primary lung tumors. Contrast Media Mol Imaging.

[CR34] Werner MK, Parker JA, Kolodny GM, English JR, Palmer MR (2009). Respiratory gating enhances imaging of pulmonary nodules and measurement of tracer uptake in FDG PET/CT. AJR Am J Roentgenol.

[CR35] Whang JS, Baker SR, Patel R, Luk L, Castro A (2013). The causes of medical malpractice suits against radiologists in the United States. Radiology.

[CR36] White CS, Pugatch R, Koonce T, Rust SW, Dharaiya E (2008). Lung nodule CAD software as a second reader: a multicenter study. Acad Radiol.

[CR37] Yang S, Weidong C, Heng H, Xiaogang W, Yun Z, Fulham MJ, Feng DD (2014). Lesion detection and characterization with context driven approximation in thoracic FDG PET-CT images of NSCLC studies. IEEE Trans Med Imaging.

